# Bifunctional CO oxidation over Mn-mullite anchored Pt sub-nanoclusters *via* atomic layer deposition†
†Electronic supplementary information (ESI) available: Catalyst preparation and characterization details, supplemental figures and tables. See DOI: 10.1039/c7sc05486f


**DOI:** 10.1039/c7sc05486f

**Published:** 2018-01-26

**Authors:** Xiao Liu, Yuanting Tang, Meiqing Shen, Wei Li, Shengqi Chu, Bin Shan, Rong Chen

**Affiliations:** a State Key Laboratory of Digital Manufacturing Equipment and Technology , School of Mechanical Science and Engineering , Huazhong University of Science and Technology , Wuhan 430074 , Hubei , People’s Republic of China . Email: rongchen@mail.hust.edu.cn; b State Key Laboratory of Materials Processing and Die and Mould Technology , School of Materials Science and Engineering , Huazhong University of Science and Technology , Wuhan 430074 , Hubei , People’s Republic of China . Email: bshan@mail.hust.edu.cn; c School of Chemical Engineering and Technology , Tianjin University , Tianjin 300072 , People’s Republic of China; d General Motors Global Research and Development , Chemical Sciences and Materials Systems Lab , 3500 Mound Road , Warren , Michigan 48090 , USA; e Institute of High Energy Physics , Chinese Academy of Sciences , Beijing 100049 , People’s Republic of China

## Abstract

CO oxidation is a widely used model system for understanding fundamental aspects of heterogeneous catalysis. While platinum (Pt) continues to be a reference material for CO oxidation catalysis, poisoning of Pt catalysts presents a critical issue that blocks reaction sites and impedes subsequent reaction steps. Fabrication of CO poison-free Pt catalysts remains a great challenge due to its CO-philic nature. Herein, we report a Pt based catalyst to effectively tackle CO poisoning by tightly anchoring Pt sub-nanoclusters onto Mn-mullite oxide (SmMn_2_O_5_) *via* atomic layer deposition. Superior CO oxidation activity has been observed with a significantly lowered light-off temperature and apparent activation energy. *In situ* diffuse reflectance infrared Fourier transform spectroscopy analysis, oxygen isotope experiments and density functional theory calculations confirm that the low-temperature activity originates from active oxygen atom sources at the bifunctional interface structure.

## Abstract 2

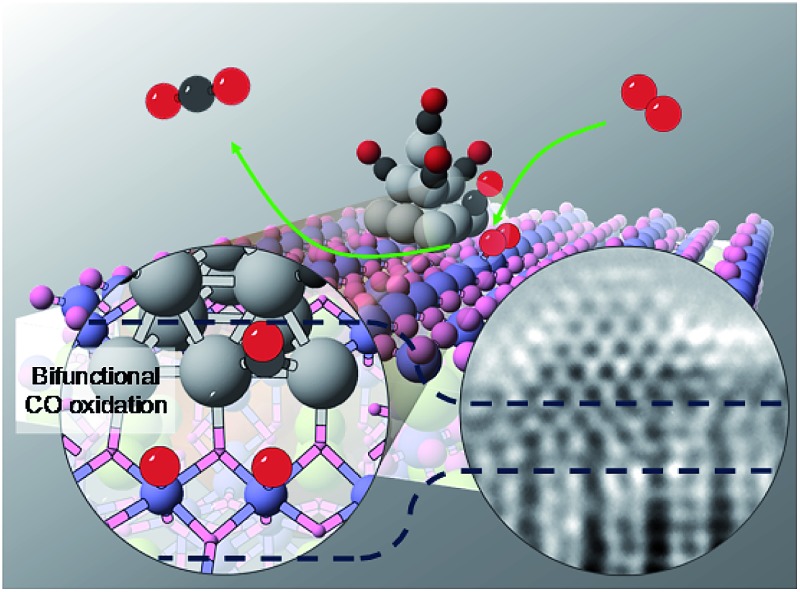
Highly dispersed Pt sub-nanoclusters are anchored on SmMn_2_O_5_ mullites *via* atomic layer deposition, and show excellent low-temperature CO oxidation activity.

## Introduction

Catalytic oxidation of CO over platinum (Pt) metal has been the subject of extensive investigations since the classical work of Langmuir, and has found important technological applications in automotive exhaust emission control and fuel cells.^1^–^3^ Overall, Pt has shown excellent performance as well as stability against oxidation and compound formation, making it the *de facto* standard reference catalyst. However, one of the critical problems that plague Pt catalysts is the well-known CO poisoning effect that limits its activity by the blockage of surface active sites and subsequent reaction steps under low temperatures.^4^–^6^ Such phenomena are quite general considering that CO will be used as a reactant or generated as a reaction intermediate in many catalytic processes such as CO oxidation and water–gas shift reactions, and in fuel cells.^7^–^9^ In view of the high dispersion (smaller Pt nanoparticles) required for catalyst applications, CO poisoning will be only more pronounced, wherein a large fraction of coordinatively unsaturated edge and vertex sites over-bind CO molecules.^10^–^13^ Iglesia *et al.* have reported that the CO molecules binding on Pt atoms with low coordination numbers are stronger than those on Pt (111), due to the greater electron donation of Pt atoms on nanocluster surfaces. The average CO binding energy on Pt nanoclusters at full coverage is also greater than that on Pt (111), due to the weaker repulsive interactions among adsorbed CO molecules.^13^ Overall, the high CO coverage will hinder the adsorption and dissociation of O_2_ molecules on Pt facets, which agrees well with our theoretical predictions.^14^

Considerable efforts have been devoted to eliminating the CO poisoning problem, primarily *via* electronic or bifunctional mechanisms. In the electronic mechanism, Pt metal is alloyed with other transitional metals to form multi-component/core–shell nanoparticles that have modified electronic structures and weakened CO binding strengths.^15^–^17^ However, the element distribution of the alloy catalysts is difficult to control and competitive adsorption cannot be completely suppressed, especially under the circumstance that other reactants interact more weakly with Pt than CO. The bifunctional mechanism, on the other hand, is a highly efficient way of mitigating CO poisoning by providing spatially separated sites for oxygen species. This is typically achieved by utilizing reducible transition metal oxide supports like CeO_2_, FeO_*x*_ and Co_3_O_4_, where the interfacial lattice oxygen of Pt–oxide composites therein can participate in the redox reactions.^18^–^22^ The oxide support must possess high bulk oxygen mobility so that oxygen species at the interface can be quickly consumed and replenished to sustain the reaction. Unfortunately, the sluggish oxygen diffusion rate would seriously limit the activity of the composite catalysts, and the oxygen transport channel may be poisoned by products or intermediates, which can lead to performance degradation.^23^–^26^ Therefore, design of a bifunctional catalyst that can supply oxygen species not limited by the bulk oxygen diffusion opens up new opportunities in the development of high-performance CO oxidation catalysts.

Herein, we report a novel Pt based composite catalyst that has the unique capability of directly dissociating gas phase oxygen molecules on the oxide site, overcoming the bulk oxygen diffusion limit for supplied oxygen species in conventional bifunctional catalysts. The composite catalyst is synthesized *via* atomic layer deposition (ALD) of Pt clusters on SmMn_2_O_5_ (SMO) mullite-type oxides, which show excellent low temperature CO oxidation activity and low apparent activation energy. *In situ* diffuse reflectance infrared Fourier transform spectroscopy (DRIFTS), ^18^O isotope-labelling experiments and density functional theory (DFT) calculations confirm that the active oxygen supplied by the bifunctional interface is the origin of the low temperature CO oxidation activity.

## Results and discussion

**Fig. 1:**
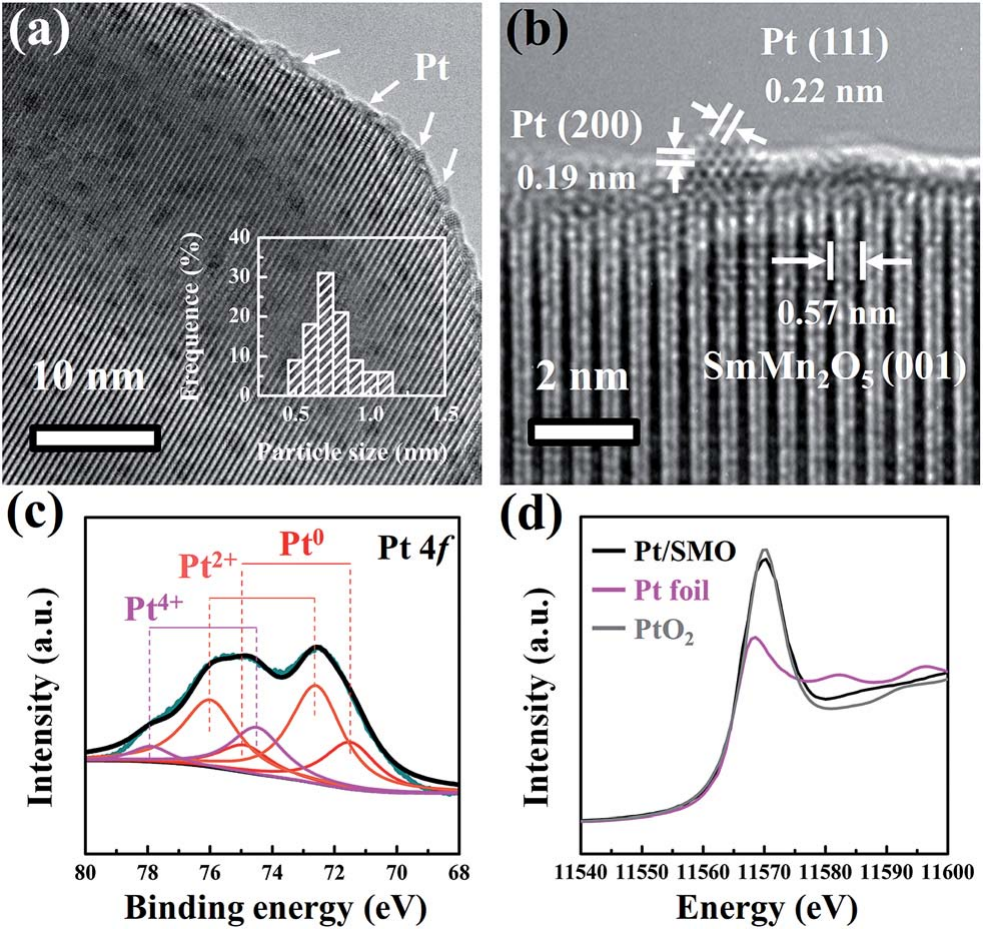
(a) TEM and (b) high resolution TEM images of the Pt/SMO catalyst are presented. The size distribution of the Pt clusters has been inserted in (a). (c) The Pt 4f XPS spectrum of the Pt/SMO catalyst. (d) The normalized Pt L_III_-edge XANES of Pt/SMO, Pt foil and PtO_2_.

The highly dispersed Pt sub-nanoclusters are synthesized *via* ALD on SMO mullite-type oxides, which are in the shape of irregular ellipsoids with an average size of about 50 nm (Fig. S1†). As shown in Fig. 1a, the Pt clusters of the as-prepared Pt/SMO catalysts are on a sub-nano scale of 0.5–0.9 nm. The bright spots in the high-angle annular dark-field scanning transmission electron microscopy (HAADF-STEM) images represent the uniformly distributed Pt clusters on the SMO supports (Fig. S2†), which agrees well with the lack of an appreciable Pt diffraction peak in the XRD pattern of Pt/SMO (Fig. S3†). The lattice fringes with a *d*-spacing of 0.57 nm in Fig. 1b are assigned to the SMO (001) planes. The lattice fringes with a *d*-spacing of 0.22 nm and 0.19 nm are assigned to the Pt (111) and (200) planes, respectively.

The XPS results (Fig. 1c) show that the main state of the Pt clusters can be attributed to oxidized Pt (Pt^2+^, 56% and Pt^4+^, 20%) and the other Pt species are in the state of metallic Pt atoms (Pt^0^, 24%). According to the morphology of Pt clusters, we assigned the oxidized Pt species to the interfacial Pt atoms bound to surface oxygen atoms and the metallic Pt atoms to the Pt atoms far away from the interface. The large amount of oxidized Pt indicates the high dispersion of Pt clusters and strong interfacial interactions. Consistent with the XPS analysis, the Pt L_III_-edge X-ray absorption near-edge structure (XANES) spectra (Fig. 1d) show that the Pt/SMO catalyst has a large amount of oxidized Pt species, with a white line intensity similar to that of the PtO_2_ sample, which further implies the strong chemical anchoring of Pt clusters on the SMO oxides. The Fourier transform extended X-ray absorption fine structure (EXAFS) spectrum (Fig. S4†) shows a strong peak at about 1.7 Å, which can be attributed to the Pt–O bonds. The fitted Fourier transform EXAFS spectra show that the fitted Pt–O bond length of Pt/SMO is about 2.007 Å and the coordination number is about 3.51 (Table S2†). The shorter Pt–O bond length and smaller Pt–O coordination number compared to those of the PtO_2_ sample also indicate the Pt/SMO interfaces as the origin of the Pt–O bonds.

**Fig. 2:**
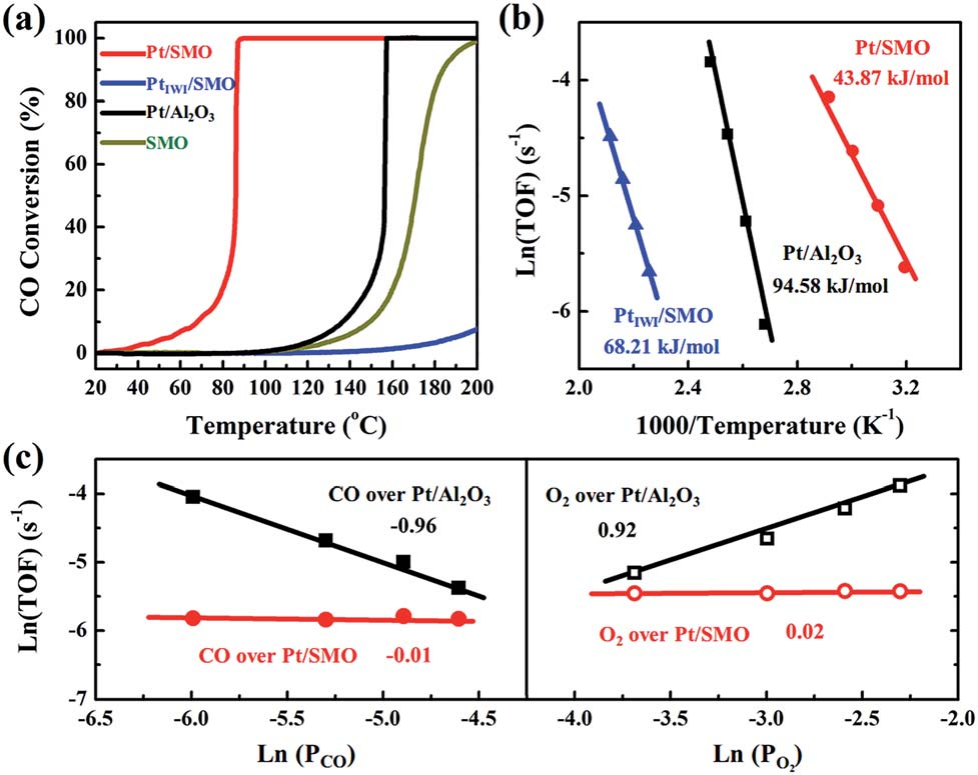
(a) The CO conversion of Pt/SMO, Pt_IWI_/SMO, Pt/Al_2_O_3_ and SMO as a function of reaction temperature. (b) The Arrhenius plots of CO oxidation rates of Pt/SMO, Pt_IWI_/SMO and Pt/Al_2_O_3_. (c) The reaction orders of CO and O_2_ over Pt/SMO and Pt/Al_2_O_3_.

A CO oxidation activity test has been performed to evaluate the CO-tolerant performance of Pt/SMO catalysts at low temperature. As shown in Fig. 2a Pt/SMO exhibits excellent low temperature CO oxidation activity with a *T*_50_ (50% CO conversion temperature) of about 86 °C, which is much lower than that of pure SMO (171 °C). It also shows excellent structural stability in multi-cycle repeated activity tests (Fig. S5†). As a control experiment, the incipient wetness impregnation method has also been used to construct the Pt/SMO composite catalyst (Pt_IWI_/SMO, Fig. S6†). Pt_IWI_/SMO exhibited poorer activity than pure SMO. The Mn 2p XPS spectra show that the concentrations of Mn^3+^ and Mn^4+^ ions of Pt_IWI_/SMO are quite different to that of pure SMO and Pt/SMO, indicating the change of surface active sites during Pt impregnation (Fig. S7†). Moreover, Pt_IWI_/SMO has a large amount of Pt^4+^ oxidation states (0.88) and the H_2_ reduction treatment can slightly enhance its activity by yielding more Pt^2+^ and Pt^0^ states (Fig. S8 and S9†). However, the enhanced activity of Pt_IWI_/SMO is still much lower than that of Pt/SMO, implying the key role of the interface structure to the high activity of the Pt/SMO catalyst. The Pt clusters have also been deposited on Al_2_O_3_ supports as a reference sample (Pt/Al_2_O_3_) by the same ALD process as for Pt/SMO. The TEM images (Fig. S10†) show that the average size of deposited Pt clusters is about 1.19 nm and there is also no characteristic Pt peak in the XRD pattern of Pt/Al_2_O_3_, indicating a high dispersion of small Pt clusters as well. The Pt clusters on Al_2_O_3_ supports are mainly in the metallic Pt state (0.62) per the Pt 4d XPS spectrum shown in Fig. S11,† which is a result of the weaker interactions between the Pt clusters and Al_2_O_3_ supports. The measured *T*_50_ of Pt/Al_2_O_3_ is about 157 °C, which is due to the CO poisoning effect at low temperatures on Pt facets.

The intrinsic behavior of Pt clusters for CO oxidation on both SMO and Al_2_O_3_ supports has been further investigated by performing kinetic tests to eliminate the thermal and diffusion effects (Fig. S12†). Arrhenius plots of reaction rates for Pt/SMO, Pt_IWI_/SMO and Pt/Al_2_O_3_ are shown in Fig. 2b. The calculated apparent activation energy of Pt/SMO (43.87 kJ mol^–1^) is smaller than that of Pt_IWI_/SMO (68.21 kJ mol^–1^) and about half of that for Pt/Al_2_O_3_ (94.58 kJ mol^–1^). Despite the high gas hourly space velocity of 120 000 mL g^–1^ h^–1^ used, Pt/SMO catalysts exhibit a much lower *T*_50_ and apparent activation energy compared with other oxide supported Pt catalysts in previous studies (Table S3†). The reaction orders of Pt/SMO and Pt/Al_2_O_3_ with respect to CO and O_2_ are presented in Fig. 2c. The reaction orders of CO and O_2_ over Pt/Al_2_O_3_ are close to –1 and 1, suggesting CO inhibition in the low temperature region and the typical Langmuir–Hinshelwood reaction mechanism.^27^,^28^ The active oxygen species during CO oxidation over Pt/Al_2_O_3_ are supplied by the O_2_ dissociation on the surface of Pt clusters, which will be inhibited by the strongly bound CO molecules at low temperatures. The reaction orders of CO and O_2_ over Pt/SMO are close to zero, indicating negligible competitive adsorption between CO and O_2_ during CO oxidation over Pt/SMO. Thus, the supply of active oxygen species for CO oxidation over Pt/SMO may be independent of the Pt sites covered by CO molecules.

**Fig. 3:**
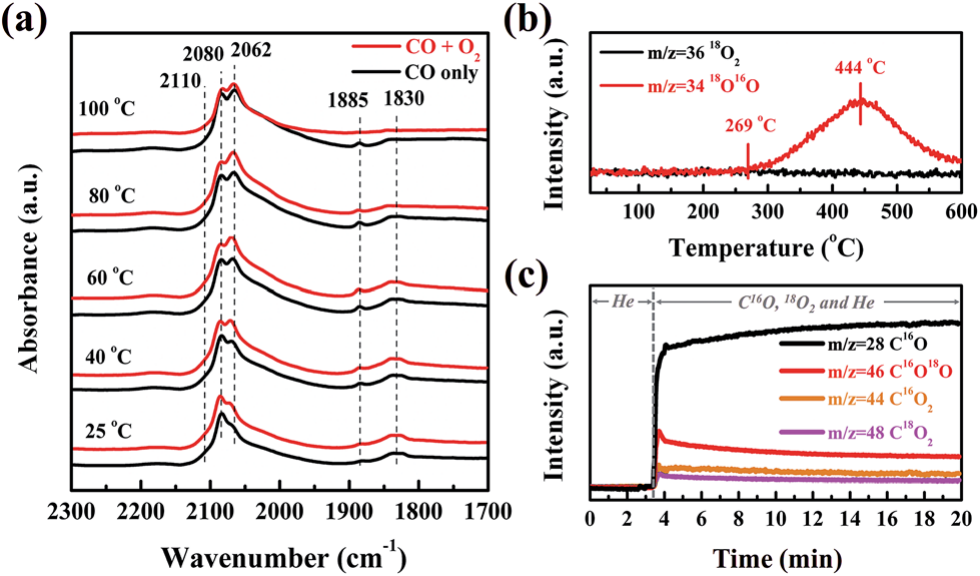
(a) *In situ* DRIFTS spectra of Pt/SMO with CO adsorption (black lines) and oxidation (red lines) at different reaction temperatures. (b) Changes of *m*/*z* = 34 and 36 as a function of temperature for the ^18^O_2_ pretreated Pt/SMO catalyst during the TPIE experiment. (c) Changes of *m*/*z* = 28, 44, 46 and 48 during CO oxidation with C^16^O and ^18^O_2_ at 80 °C as a function of time for the Pt/SMO catalyst after ^16^O_2_ pretreatment.

In order to clarify the origin of the active oxygen species in the CO oxidation over Pt/SMO, the CO adsorption and O_2_ dissociation behaviors have been investigated. As shown in Fig. 3a, the *in situ* DRIFTS spectra show that there are three sets of peaks after CO molecular adsorption (black lines) on Pt/SMO at room temperature (25 °C), which are assigned to the CO molecules adsorbed at the bridge sites (1830 cm^–1^ and 1885 cm^–1^), the top sites (2060 cm^–1^ and 2080 cm^–1^, as well as the broad shoulder (1950–2050 cm^–1^)) and the oxidized Pt clusters (2110 cm^–1^).^29^–^31^ Only the bridge-bonded CO molecules (1830 cm^–1^) will be desorbed as the temperature increases to 100 °C. Upon introduction of the oxygen gas feed, the bridge-bonded CO molecules can be observed to react with the injected O_2_ at 80 °C (red line), while the linear-bonded CO molecules are very stable on the Pt clusters. Moreover, after all of the bridge-bonded CO molecules have reacted with O_2_ at 100 °C, large amounts of linear-bonded CO molecules remain on Pt clusters. Therefore, it can be deduced that the bridge-bonded CO molecules at the interface are the primary active source for CO oxidation at low temperature. As a comparison, the *in situ* DRIFTS spectra of CO oxidation over Pt/Al_2_O_3_ (Fig. S13†) show that the injected O_2_ will not react with the adsorbed CO molecules when the temperature is below 140 °C. When the temperature reaches 160 °C (higher than *T*_50_), the bridge-bonded and linear-bonded CO molecules on the Pt clusters both disappear rapidly, showing no apparent selectivity for the oxidation of these two types of CO molecule.

To further identify the source of active oxygen during CO oxidation, an isotope-labelling method was implemented. The temperature programmed isotope exchange (TPIE) experiment for ^18^O_2_ pretreated Pt/SMO shows that the initial temperature of hetero-exchange between the gas phase and solid surface is about 269 °C (Fig. 3b). There is no obvious hetero-exchange at low temperatures, indicating the low activity of lattice oxygen species for low temperature reactions. Specifically, we have utilized an isotope-labelling method to study the source of active oxygen for CO oxidation over the ^16^O_2_ pretreated Pt/SMO catalyst at 80 °C. As shown in Fig. 3c, after C^16^O and ^18^O_2_ are injected into the system, the mass intensity of C^16^O will be sharply increased. Simultaneously, the increased mass intensity of C^16^O_2_, C^16^O^18^O and C^18^O_2_ (*m*/*z* = 44, 46 and 48) can be related to the CO oxidation reaction at 80 °C. Among the three product species, the trace amount of C^18^O_2_ is almost negligible. Depending on the source of oxygen atoms, C^16^O_2_ and C^16^O^18^O can be assigned to the process of C^16^O reacting with lattice oxygen (^16^O) and dissociated oxygen (^18^O), respectively. Fig. 3c demonstrates that C^16^O^18^O is the dominant species in the CO_2_ products, confirming that dissociated oxygen rather than lattice oxygen is the main source of active oxygen for low temperature CO oxidation. As most Pt sites on Pt clusters have been poisoned by CO molecules, the dissociation takes place on the active sites of the SMO surface at the Pt/SMO interface.

**Fig. 4:**
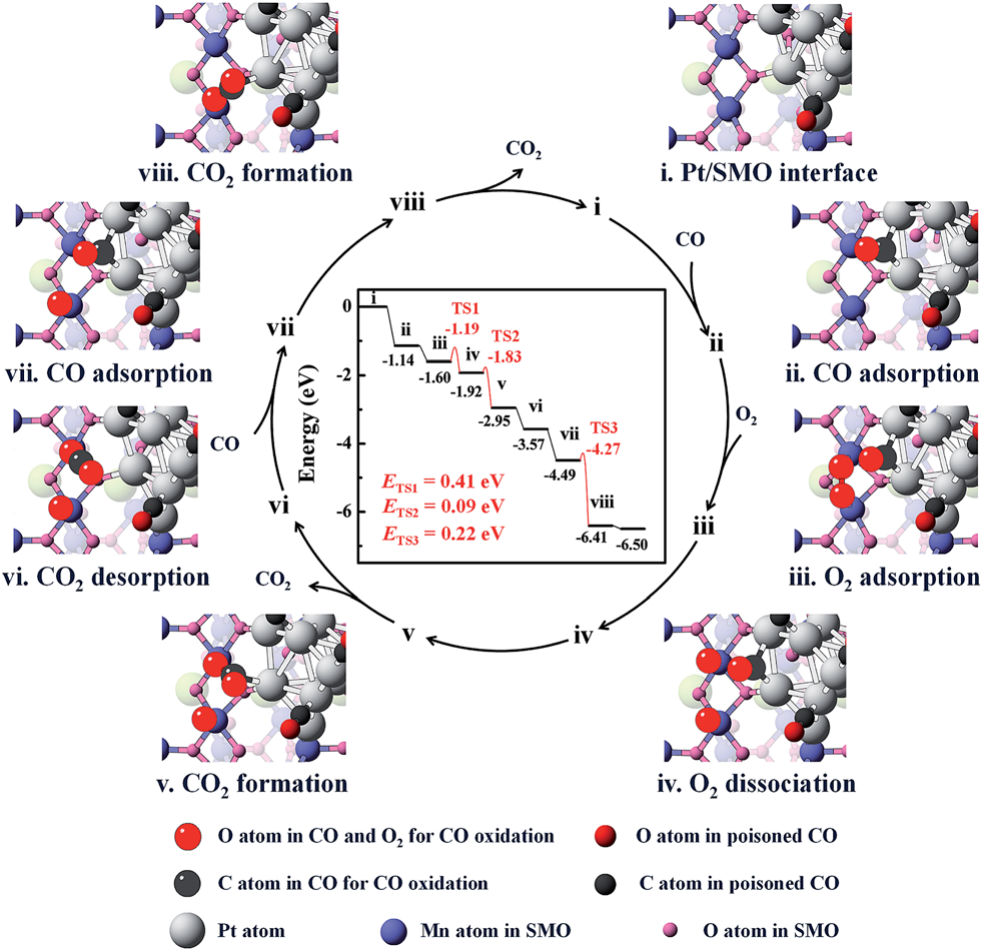
The energetic route of CO oxidation at the interface of Pt/SMO. The catalytic cycle can be summarized as 2CO + O_2_ → 2CO_2_.

DFT calculations were employed to elucidate the dissociation process of O_2_ at the Pt/SMO interface. The pyramidal shaped Pt_10_ cluster has been selected considering that it is the most stable cluster in the gas phase.^32^,^33^ Interface geometry relaxation of the structure shows that Pt_10_ has been effectively anchored on the SMO (010) facet with an exposed Mn_2_ dimer (Fig. S14†), which is a thermodynamically stable species and has been shown to possess high activity towards O_2_ dissociation in our previous theoretical study.^34^ CO adsorption on the exposed Mn_2_ dimer is very weak (–0.31 eV) and the binding strength of CO on Pt at the interface is much weaker than that on Pt sites far away from the interface (Fig. S15†). This can be attributed to the more-vacant d orbitals of interfacial Pt atoms due to outward charge transfer to the SMO support (Fig. S16†), which is consistent with our XPS results and other Pt/oxide systems in previous studies.^31^,^35^ Considering the strong poisoning of CO (<–2 eV) on Pt atoms away from the interface, we have constructed a CO covered Pt/SMO model (Fig. S17†) as the initial state for the catalytic cycle calculation. Competitive adsorption energy analysis of CO and O_2_ at the Pt/SMO interface (Fig. S17†) shows that CO adsorption prefers the bridged sites of interfacial Pt, and O_2_ prefers the Mn_2_ dimer, creating an effective spatially separated adsorption for the two reactants. The complete reaction route of CO oxidation at the Pt/SMO interface is illustrated in Fig. 4. The CO molecule initially adsorbs on the Pt interfacial site with an adsorption energy of –1.14 eV and the O_2_ molecule adsorbs on the Mn_2_ dimer with an adsorption energy of –0.45 eV. Subsequently, the adsorbed oxygen will dissociate into active oxygen atoms on the Mn_2_ site with a low barrier energy of 0.41 eV. CO molecules can react with active oxygen atoms sequentially and form CO_2_ molecules with barrier energies of 0.09 eV and 0.22 eV, respectively. During the whole reaction route, there is no competitive adsorption between CO and O_2_ at the interface, and the CO adsorbed on the Pt sites cannot suppress the O_2_ adsorption and dissociation steps on the Mn dimers, which is consistent with the zero-order rate results for the Pt/SMO catalyst. Overall, the O_2_ dissociation step on the Mn_2_ component is the rate determining step (RDS) of CO oxidation over the Pt/SMO interface with a barrier energy of 0.41 eV (39.51 kJ mol^–1^), which is comparable to the experimentally measured apparent activation energy. Comparing the barrier energies of O_2_ dissociation and CO_2_ formation of Pt/SMO to that at the interface of Pt clusters and other oxides in previous studies (Table S4†), Pt/SMO shows a relatively low reaction barrier and can be more active towards CO oxidation. This indicates that the Pt/SMO interface plays a key role in bifunctional CO oxidation catalysis.

## Conclusions

In conclusion, a bifunctional Pt/SMO interfacial structure is synthesized by tightly anchoring Pt sub-nanoclusters on SMO oxides, which provides spatially separated sites for CO and O_2_. With CO binding to Pt and effective O_2_ dissociation on the Mn_2_ dimer, the interface serves as an efficient poison-free CO oxidation site. The proposed catalytic reaction mechanism of CO oxidation over Pt/SMO shows that the high activity of O_2_ dissociation at the bifunctional interface is the key to the low temperature CO oxidation activity. Our work on the bifunctional catalyst removes the lattice oxygen diffusion limit and sheds light on the design of new poison-free CO oxidation catalysts.

## Conflicts of interest

There are no conflicts to declare.

## Supplementary Material

Supplementary information
