# Supplementary material for: Bifunctional CO oxidation over Mn-mullite anchored Pt sub-nanoclusters via atomic layer deposition
Source: Chem Sci. 2018 Jan 26;9(9):2469–73. doi: 10.1039/c7sc05486f (PMC5909126; doi:10.1039/c7sc05486f)
Supplement: Supplementary file 1 [file SC-009-C7SC05486F-s001.pdf]

Electronic Supplementary Material (ESI) for Chemical Science.
This journal is © The Royal Society of Chemistry 2018

Supplemental information

Bifunctional CO oxidation over Mn-mullite anchored Pt sub-nanoclusters via atomic layer deposition

Xiao Liu, Yuanting Tang, Meiqing Shen, Wei Li, Shengqi Chu, Bin Shan* & Rong Chen*

*Corresponding authors: rongchen@mail.hust.edu.cn (Rong Chen); bshan@mail.hust.edu.cn (Bin Shan).

The file includes:

Experimental details

Figures S1-S17

Tables S1-S4

References

Experimental details

Catalysts preparation. The coprecipitation method was chosen to prepare the $SmMn_2O_5$ (SMO) mullite oxide as reported in previous studies.[1,2] Briefly, $Sm(NO_3)_3 \cdot 6H_2O$, $Mn(NO_3)_2$ and Pluronic F127 (surfactant) with the appropriate amounts were dissolved by deionized water, which were purchased from Aladdin. Then, tetramethylammonium hydroxide was used to tune the pH of solution to 9 ~ 10 and H_2O_2 was added to the solution to reduce the Mn ions in the following step. The mixture was stirred for two hours at room temperature and filtered to get the SMO precursors. At last, the SMO products were prepared by performing the over-night drying at 100 ºC and calcination treatments on the precursors. The dried SMO precursors were firstly calcined at 500 ºC for 8 h, and then the calcination treatment at 800 ºC is followed for 8 h. The ramp rates for both calcination treatments are 5 ºC/min.

The Pt/SMO composite catalyst was prepared by atomic layer deposition (ALD) method with the precursors of trimethyl(methylcyclopentadienyl)platinum (98%, Sigma-Aldrich) and 11 vol. % O_3 balanced by O_2. The home-made ALD chamber based on the principle of fluidized bed was kept at 200 ºC, which was used in our previous study.[3] About 200 mg of SMO supports were held into the powder holder during Pt ALD. The stainless steel bottle containing Pt precursor was kept at 65 ºC to increase its vapor pressure. The pretreatment process of 30 min with 500 mL/min of O_3 has been performed to fluidize the SMO supports sufficiently. One cycle Pt ALD was performed to prepare the Pt/SMO catalyst with the long pulse and purge time of 200 s and 200 s, respectively. The same ALD recipe of Pt/SMO was also used for the preparation of Pt/Al_2O_3 reference.

The incipient wet-impregnation method was used to prepare Pt_{IWI}/SMO catalyst. Firstly, 1.00 g of SMO supports was immersed in 1 mL $Pt(NO_3)_2$ solution that contained 0.02 g Pt. The mixture was continuously stirred about 15 min, and dried at 60 ºC for 6 hours after $Pt(NO_3)_2$ solution was totally adsorbed by SMO supports. Then, the dried precursors were calcined at 450 ºC for 4 hours to get the Pt_{IWI}/SMO catalyst.

Characterizations. The X-ray diffraction (XRD) patterns were recorded by the PANalytical X'Pert Pro with a Cu Kα1 radiation source. The Pt mass loading of catalysts were measured by inductively coupled plasma atomic emission spectrometer (ICP-OES, Optima 4300 DV). Transmission electron microscopy (TEM) and high-resolution TEM observations were performed by Tecnai G2 F30 electron microscope (FEI) to characterize the morphology of catalysts and interfacial structure. The valence states of deposited Pt clusters were characterized by X-ray photoelectron spectrometer (XPS, AXIS-ULTRA DLD-600W), the binding energies of which were calibrated according to the C1*s* at 284.8 eV. The X-ray absorption fine structure (XAFS) measurements were carried out at the 1W1B beamline of Beijing synchrotron radiation facility. The signals at the Pt L_{III}-edge (11368-12463 eV) of Pt/SMO were collected. The Pt L_{III}-edge of Pt foil and PtO_2 were tested as references. The Pt k^2-weighted Fourier transformed extended X-ray absorption fine structure (EXAFS) spectrum of Pt/SMO was analyzed with the Pt foil and PtO_2 modes by using Demeter program.[4] The parameters such as coordinated number (N), bond length (R, Å), Debye-Waller factor (σ^2, Å^2) and shift in the edge energy (ΔE_0, eV) were fitted.

The *in situ* diffuse reflectance infrared Fourier transform spectroscopy (DRIFTS) spectra of CO adsorption at different temperature were collected by wide band mercury cadmium telluride (MCT-B) detector deployed on Nicolet iS50 FTIR spectrometer (ThermoFisher Scientific). The DRIFTS cell with a KBr window was connected to a reaction gas system with ultrahigh purity N_2, 1% vol. CO balanced by N_2, and 2% vol. O_2 balanced by N_2. The samples were firstly pretreated at 200 ºC by 30 ml/min of N_2 for 30 min, and then the background spectra were collected after the samples cooling to room temperature. Note that, the samples were heated to a specified temperature (25 ºC, 40 ºC, 60 ºC, 80 ºC, 100 ºC for Pt/SMO and 25 ºC, 100 ºC, 120 ºC, 140 ºC, 160 ºC for Pt/Al_2O_3) by 30 ml/min of N_2. For the CO adsorption at the specified temperature, continuous 15 ml/min of 1% vol. CO and 15 ml/min N_2 were introduced to the DRIFTS cell for 30 min, and then the spectra were collected. Similarly, the collection of spectra for CO oxidation were carried out after introducing continuous 15 ml/min of 1% vol. CO and 15 ml/min of 2% vol. O_2 to the DRIFTS cell

for 30 min. Then, 30 ml/min of N_2 was introduced for 30 min to purge the reaction gas before the next heating process.

The isotope-labelling experiments were carried out by VDSorb-91x chemisorption analyzer. For temperature programed isotope exchange (TPIE) experiment, 25 mg of Pt/SMO loaded into the U-type quartz tube reactor was pretreated by 50 ml/min of 1% $^{18}O_2$ (balanced by He) at 400 ºC for 60 min. Then the signals of m/e = 34 and 36 ($^{16}O^{18}O$ and $^{18}O_2$) were monitored by the AMETEK® quadrupole mass spectrometer (QMS) from room temperature to 600 ºC with the continuous 50 ml/min of 1% $16O_2$ (balanced by He). For the isothermal CO oxidation, 25 mg of Pt/SMO was firstly pretreated by 50 ml/min of 1% $16O_2$, and then the sample was cooled to 80 ºC with He. Subsequently, 50 ml/min of 1% $18O_2$ and 0.5% $C^{16}O$ (balanced by He) were introduced to the reactor and the signals of m/e = 28, 44, 46 and 48 ($C^{16}O$, $C^{16}O_2$, $C^{16}O^{18}O$ and $C^{18}O_2$) were monitored by QMS.

Activity evaluation. The catalytic activity of catalysts for CO oxidation was also evaluated by VDSorb-91x chemisorption analyzer. 50 mg of catalysts were loaded into the U-type quartz tube reactor. 100 mL/min of feed gas consisted by 1% vol. CO, 10% vol. O_2 and N_2 flowed through the reactor for catalytic reaction tests, which was corresponding to the space velocity of 120000 mL (g^{-1} h^{-1}). The reactor was heated from room temperature to 200 ºC with the rate of 2 ºC/min. The partial pressures of CO and CO_2 in the tail gas were monitored by the *in situ* HPR-20 mass spectrometer. The CO conversion (X_{CO}) was calculated by:

$$X_{CO} = \frac{p_{CO_2}^{T} - p_{CO_2}^{start}}{p_{CO_2}^{end} - p_{CO_2}^{start}} \times 100\% \quad (1)$$

where $p_{CO_2}^{start}$ $p_{CO_2}^{end}$ and $p_{CO_2}^{T}$ are the partial pressure of CO_2 in the tail gas before CO oxidation, after the total conversion of CO and at a reaction temperature (T), respectively, which had been calibrated by the portable emission analyzer (MEXA-584L, Horiba). The turn-over frequency (TOF) of the catalysts at a reaction temperature was calculated by:

$$\mathrm{TOF} = \frac{\frac{P \times V}{R \times T} \times w_{CO} \times X_{CO}}{n_{Pt} \times w_{surf}} \quad (2)$$

where P, V and R were the pressure of feed gas, the flow rate of feed gas and universal gas constant, which were equal to 101325 Pa, 1.67*10^{-6} m^3/s and 8.134 J mol^{-1} s^{-1}, respectively. w_{CO} and X_{CO} were the vol. percentage (1%) of CO and the CO conversion at the temperature (T). n_{Pt} was the mass of Pt nanoparticles in mole, which was calculated by the mass loading of Pt. w_{surf} was the percentage of surface Pt atoms compared with the total Pt atoms, which was estimated by the average size of Pt clusters.[5] The detailed data had been summarized in Table S1. In order to eliminate the thermal and diffusion effects, the kinetics tests were performed with CO conversion less than 15 % at the reaction temperature by decreasing the mass of catalysts. The activation energies (E_a) of catalysts were calculated according to the slope of the Arrhenius plots. During the reaction orders tests, the feed gas was changed to 0.25~1.0% vol. CO and 2.5~10% vol. O_2 balanced by N_2 with a fixed space velocity of 120000 mL (g^{-1} h^{-1}).

Density functional theory calculations. All spin-polarized density functional theory (DFT) calculations were carried out by using the Vienna Ab initio Simulation Package (VASP).[6,7,8] The antiferromagnetic state was chosen as the ground state of SMO with the optimized lattice constants of 2a = 1.464 nm, b = 0.859 nm, c = 0.568 nm, which were consistent with previous experimental and theoretical studies.[9,10] The exchange and correlation energy was described by Perdew-Burke-Ernzerhof (PBE)[11] functional and the ionic cores were treated by projector augmented wave (PAW)[12] method. The energy cutoff of plane wave basis was set to 400 eV. The atomic structural optimization was not converged until the Hellmann-Feynman force on each atom is smaller than 0.05 eV/Å. The Monkhorst-Pack[13] *k*-mesh was set to 3 × 5 × 7 for the structure optimization of a 2 × 1 × 1 SMO supercell. The SMO (010) slab with the thickness of about 10 Å was used to simulated the SMO oxide supports. The thickness of vacuum layer was set to 15 Å to avoid the interactions between two periodic slabs. The *k*-mesh was set to 2 × 2 × 1 and the energy

difference for total energy was smaller than 5 meV for a finer k-mesh. The minimum energy paths of O_2 dissociation and CO_2 formation steps were calculated by using the climbing image nudged elastic band (CI-NEB)[14] method. During the CI-NEB calculations, six intermediate images were used to make sure the accuracy of results.

Figures S1-S17

Fig. S1

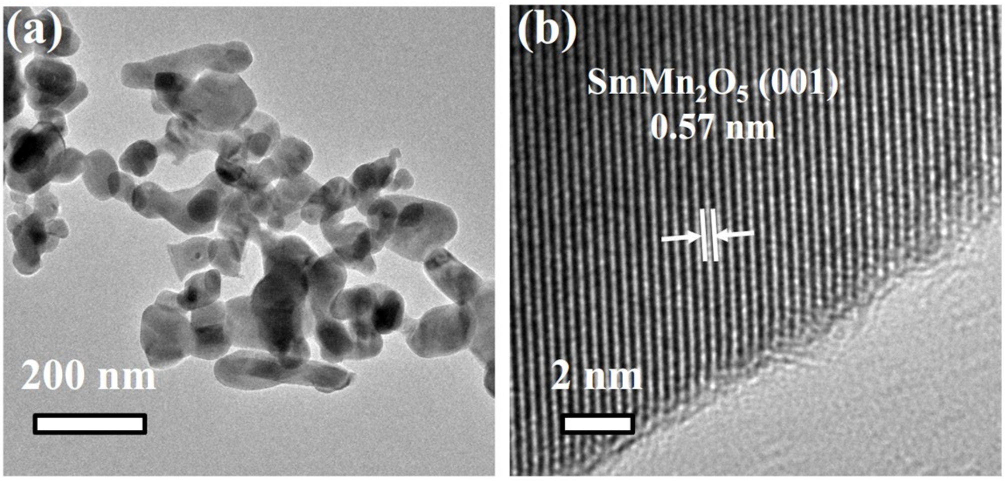

Fig. S1 The TEM images of SMO oxide supports prepared by coprecipitation method.

Fig. S2

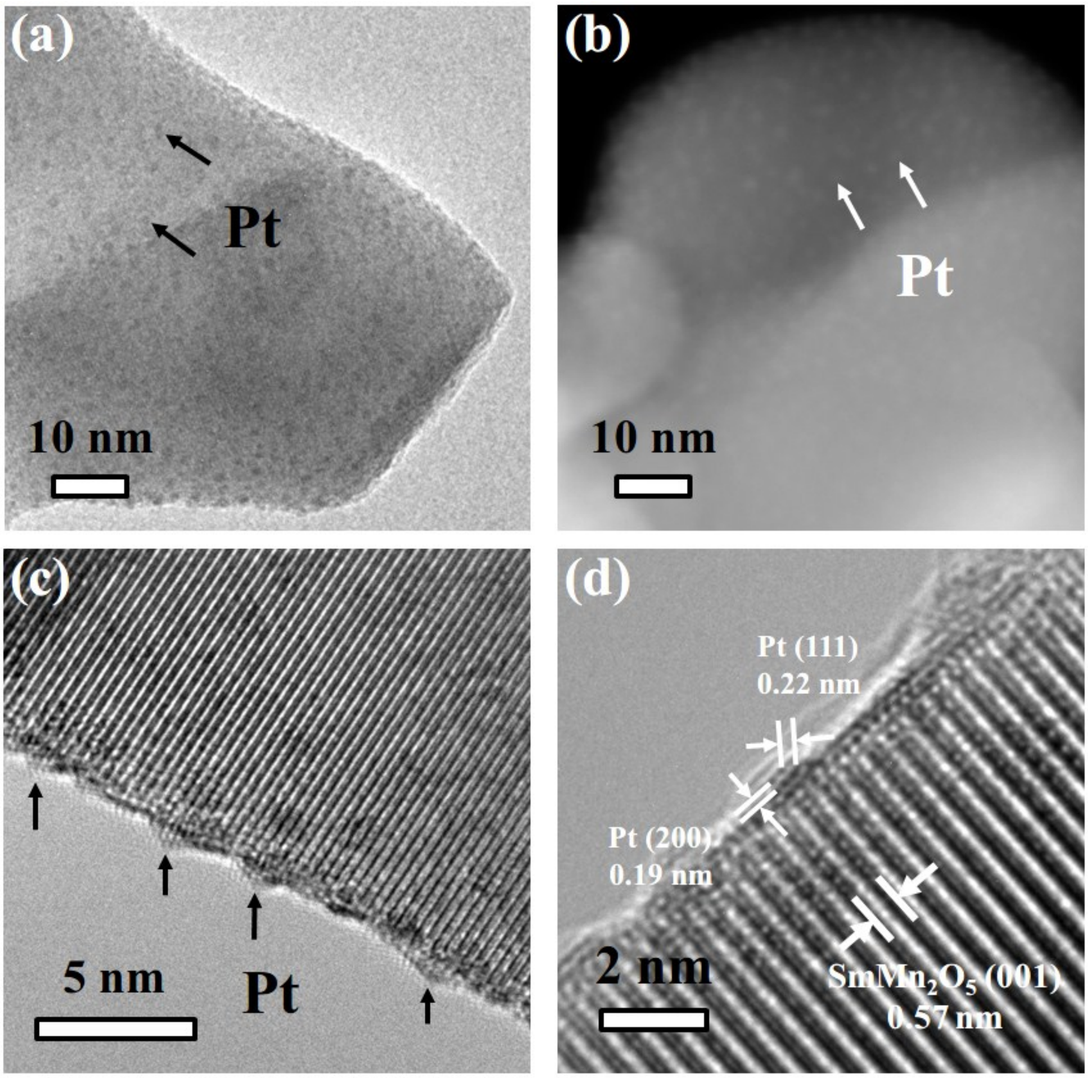

Fig. S2 (a) TEM and (b) high-angle annular dark-filed scanning transmission electron microscopy (HAADF-STEM) images of Pt/SMO. (c) and (d) are the high resolution TEM images of Pt/SMO, which focus on the Pt clusters at the edge of SMO oxide supports.

Fig. S3

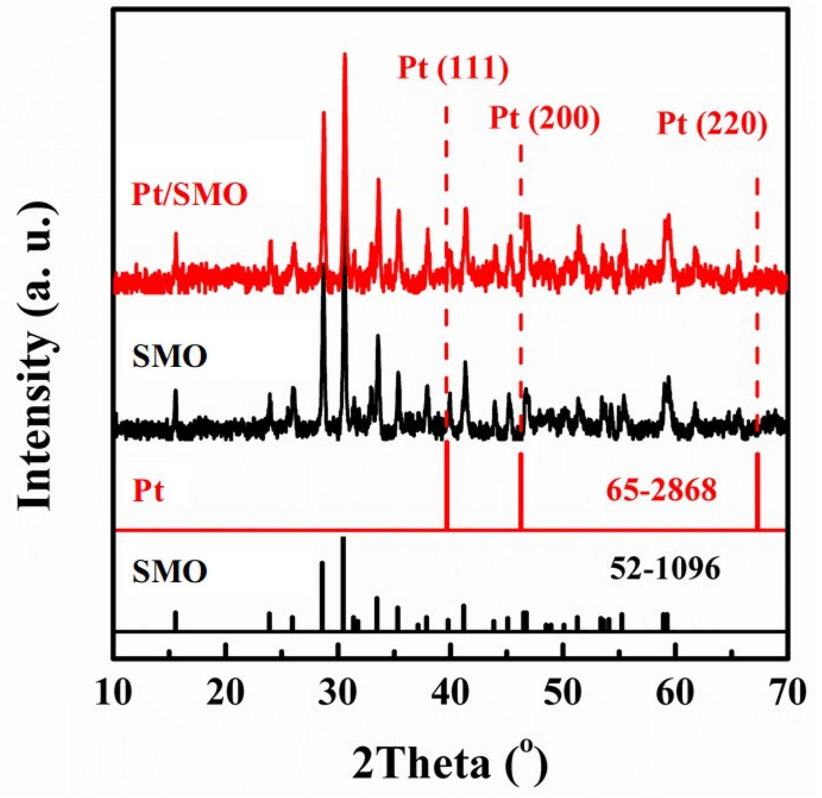

Fig. S3 The XRD patterns of SMO and Pt/SMO. It is difficult to observer Pt's diffraction peaks for Pt/SMO catalyst, which may due to the high dispersion and small size of Pt clusters.

Fig. S4

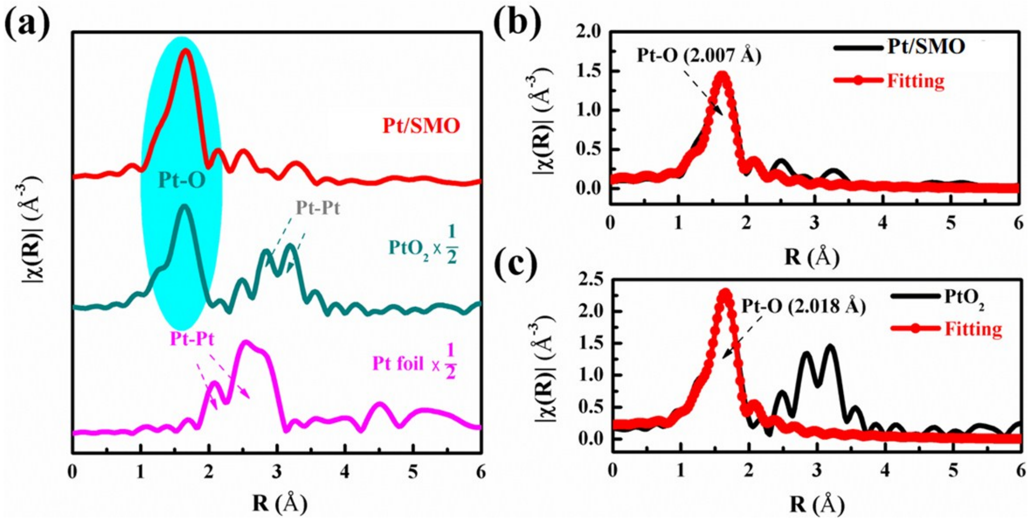

Fig. S4 (a) The Fourier transformed EXAFS spectra of Pt/SMO, PtO_2 and Pt foil. The fitting with Pt-O bond of Fourier transformed EXAFS spectra of (b) Pt/SMO and (c) PtO_2. The detailed fitting parameters have been listed in Table S2.

Fig. S5

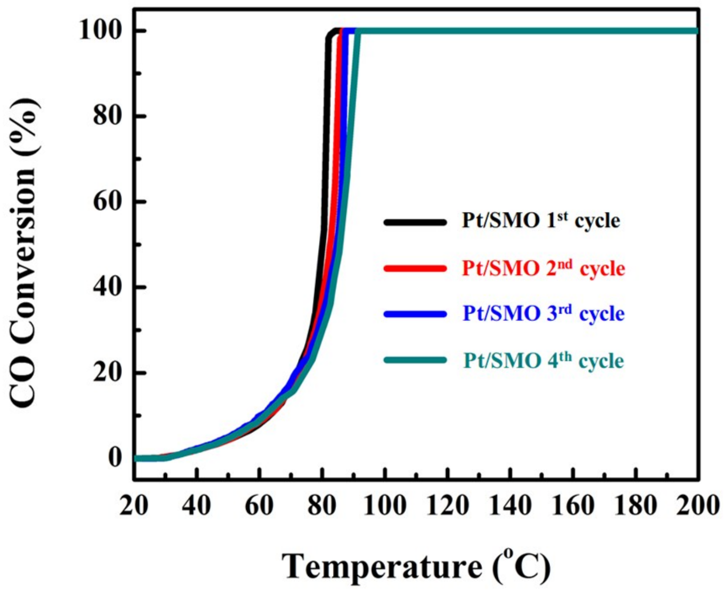

Fig. S5 CO conversion curves of Pt/SMO catalyst for four cycles. The changes of T_{50}s for the four curves are less than 5 ºC, which can indicate the structural stability of Pt/SMO during the activity test.

Fig. S6

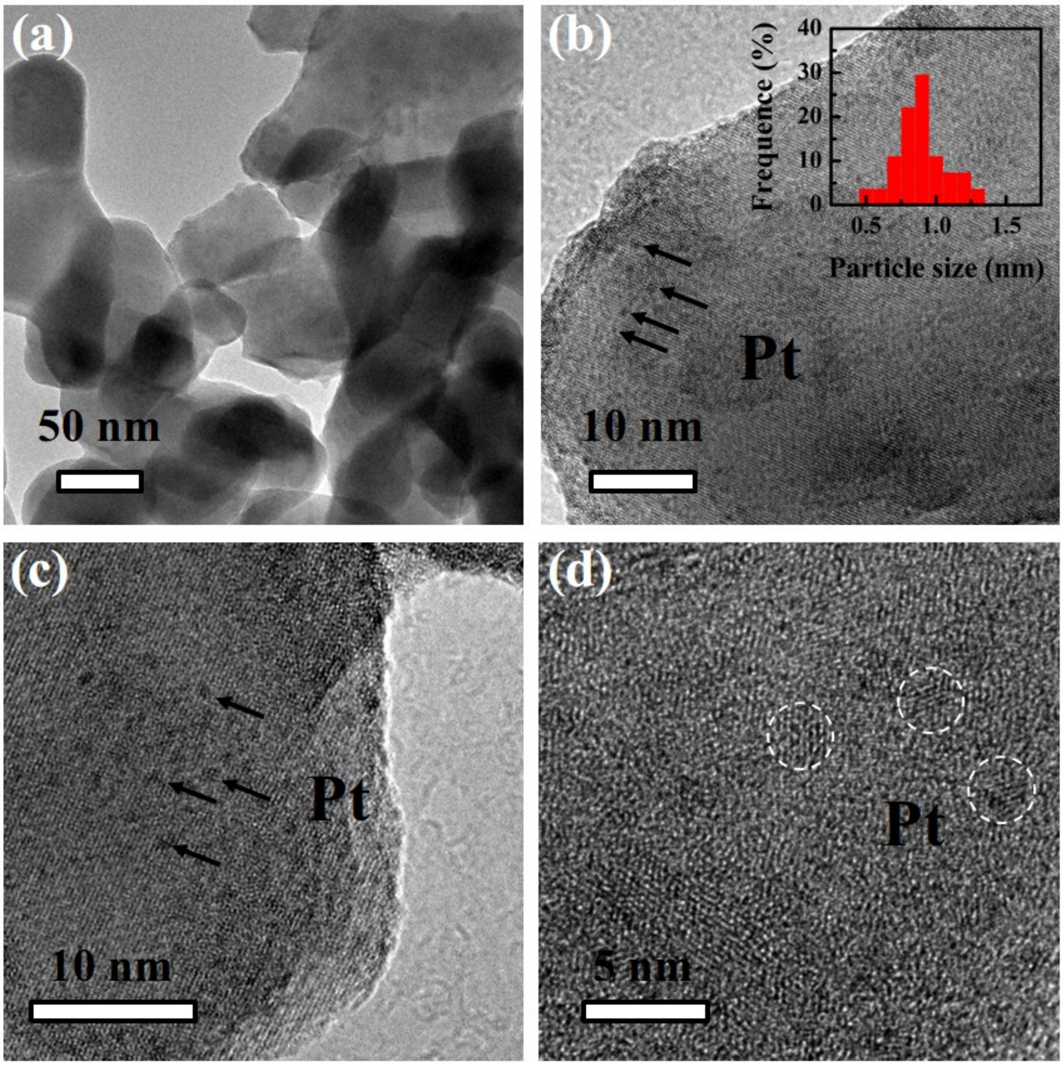

Fig. S6 The TEM images of Pt_{IWI}/SMO prepared by incipient wetness impregnation method. It is difficult to observe the Pt clusters at the edge of SMO supports and the interface structure between Pt clusters and SMO supports cannot be well controlled. The undulating edge of SMO supports may imply the change of surface structures in the preparation process of supported Pt clusters, which can be further demonstrated by the XPS results in Fig. S7.

Fig. S7

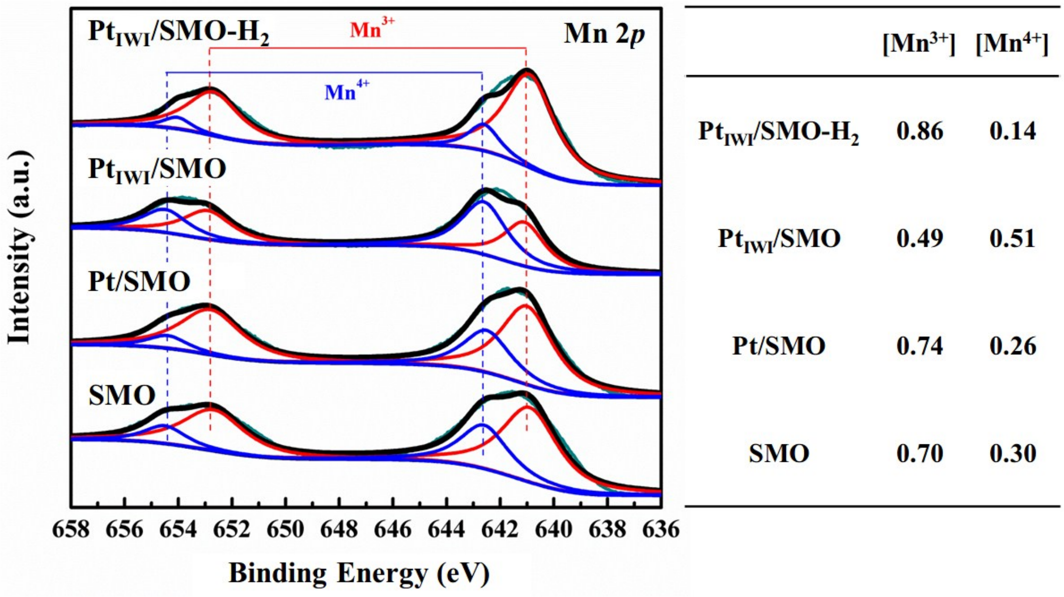

	[Mn^{3+}]	[Mn^{4+}]
Pt$_{IWI}$/SMO-H$_2$	0.86	0.14
Pt$_{IWI}$/SMO	0.49	0.51
Pt/SMO	0.74	0.26
SMO	0.70	0.30

Fig. S7 Mn 2*p* XPS spectra of SMO, Pt/SMO, Pt$_{IWI}$/SMO and Pt$_{IWI}$/SMO after H_2 reduction (Pt$_{IWI}$/SMO-H_2). The H_2 reduction treatment is performed at 300 ºC for 1 h using 10 vol. % H_2 balanced by Ar. The concentrations of Mn^{3+} and Mn^{4+} are calculated by the integrated areas of their corresponding peaks. After Pt clusters are deposited on SMO by ALD method, there is only slight change of the concentrations of Mn^{3+} and Mn^{4+} ([Mn^{3+}] and [Mn^{4+}]). However, the incipient wet-impregnation method can cause significant changes of [Mn^{3+}] and [Mn^{4+}], which can indicate the surface structure's change of SMO supports.

Fig. S8

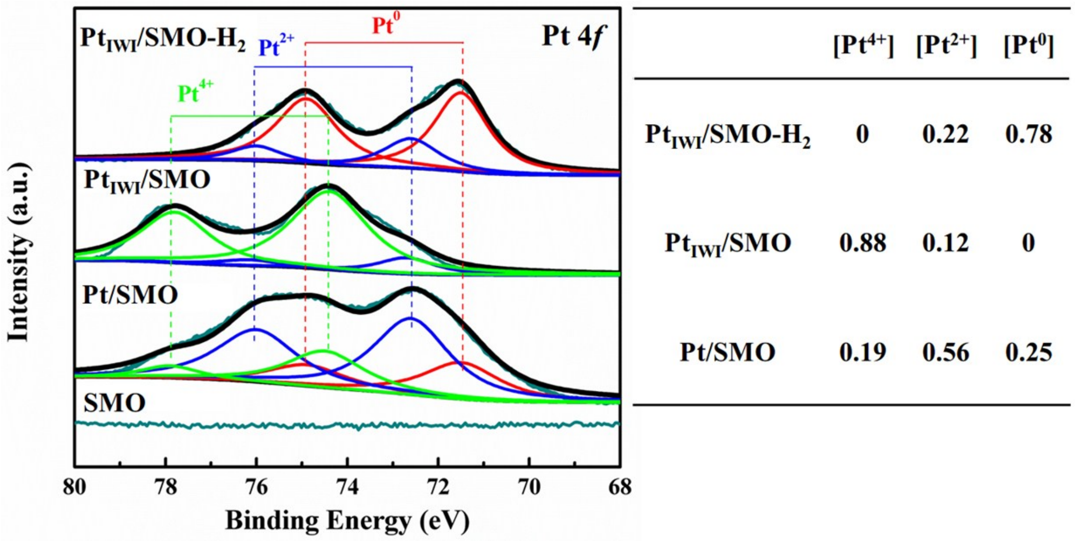

	[Pt^{4+}]	[Pt^{2+}]	[Pt^0]
Pt$_{IWI}$/SMO-H$_2$	0	0.22	0.78
Pt$_{IWI}$/SMO	0.88	0.12	0
Pt/SMO	0.19	0.56	0.25

Fig. S8 Pt 4*f* XPS spectra of SMO, Pt/SMO, Pt$_{IWI}$/SMO and Pt$_{IWI}$/SMO-H$_2$. The concentrations of Pt^{4+}, Pt^{2+} and Pt^0 have been listed in the right table. It is obviously that H$_2$ reduction treatment can yield more Pt^{2+} and Pt^0 states for Pt$_{IWI}$/SMO catalyst.

Fig. S9

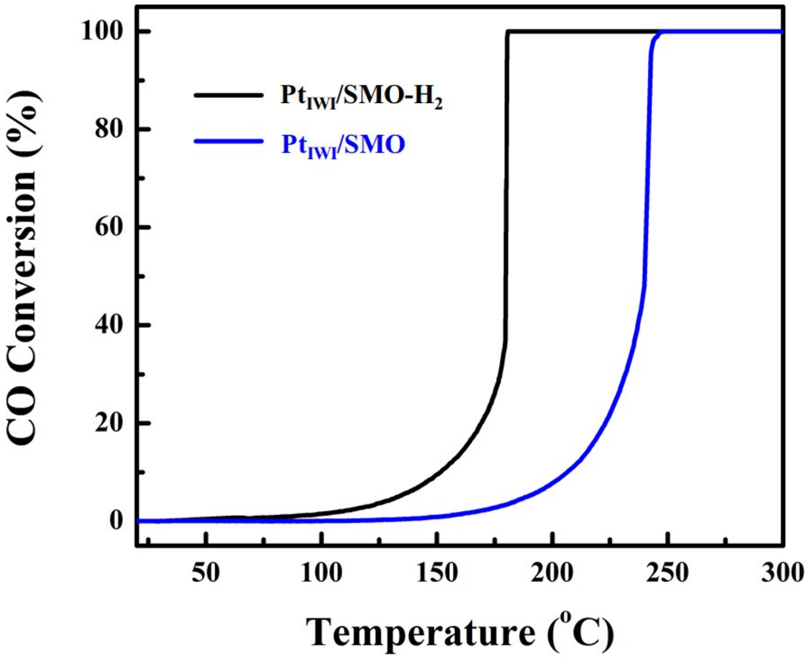

Fig. S9 The CO conversion curves of Pt$_{IWI}$/SMO-H_2 and Pt$_{IWI}$/SMO. The results show that the activity of Pt$_{IWI}$/SMO can be enhanced after H_2 reduction treatment, which may due to the change of Pt clusters' chemical states. However, the T_{50} of Pt$_{IWI}$/SMO-H_2 is 180 ºC, which is still much higher than that of Pt/SMO.

Fig. S10

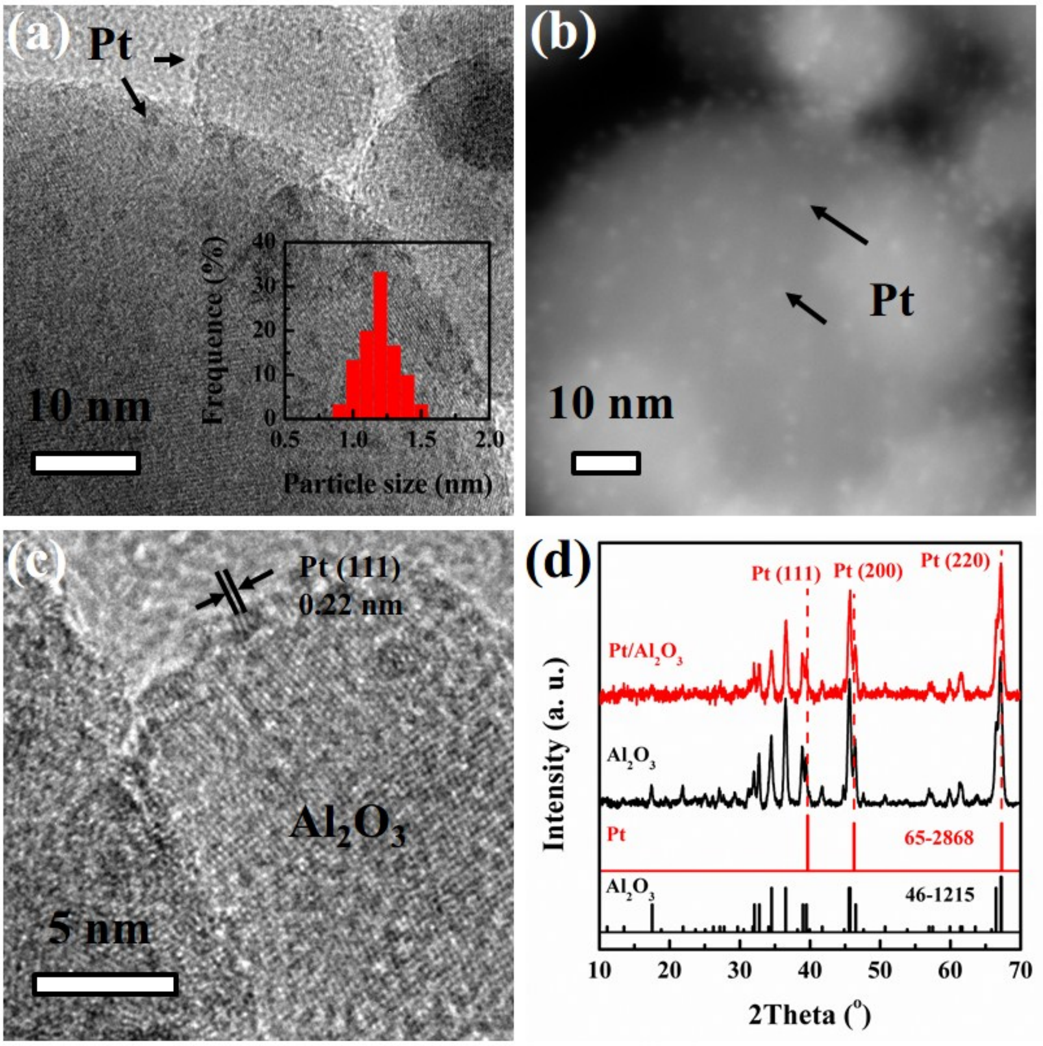

Fig. S10 (a) TEM, (b) HAADF-STEM and (c) high resolution TEM images of Pt/Al_2O_3. The inserted figure in (a) shows the size distribution of Pt clusters on Al_2O_3 supports. (d) The XRD patterns of Al_2O_3 supports and Pt/Al_2O_3.

Fig. S11

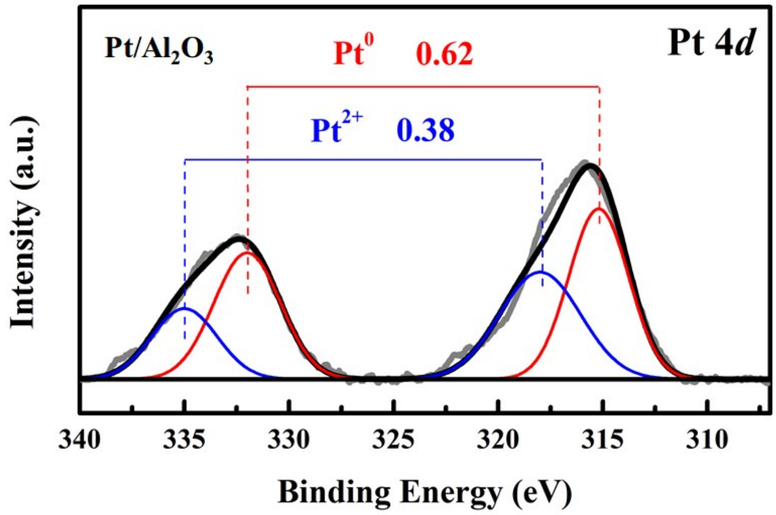

Fig. S11 The Pt 4*d* XPS spectrum of Pt/Al_2O_3 are tested due to the mutual interference between Pt 4*f* and Al 2*p* spectra. The labelled concentrations of Pt^0 (0.62) and Pt^{2+} (0.38) indicate that Al_2O_3 supported Pt clusters are mainly in metallic Pt state, which can imply the weak metal-support interaction.

Fig. S12

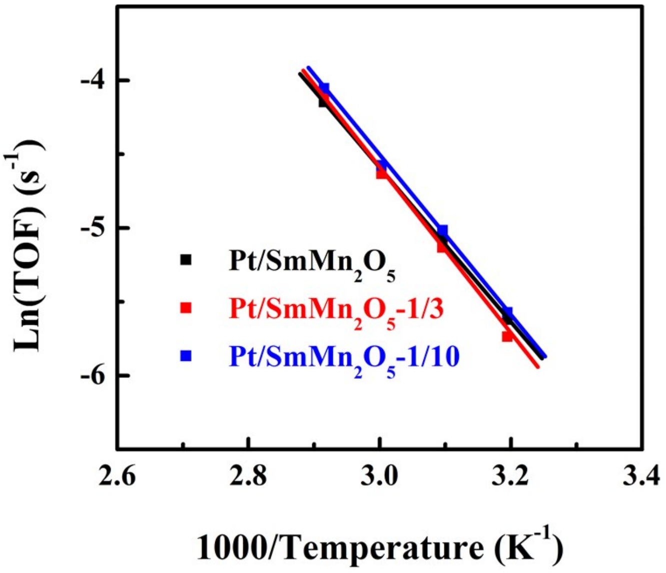

Fig. S12 The CO oxidation rates of catalysts. The pure catalyst (Pt/$SmMn_2O_5$) or diluted with inert quartz sand have been tested. Two different mass ratios between catalyst and quartz sand have been used, which are 1:3 and 1:10 (labeled as Pt/$SmMn_2O_5$-1/3 and Pt/$SmMn_2O_5$-1/10).

Fig. S13

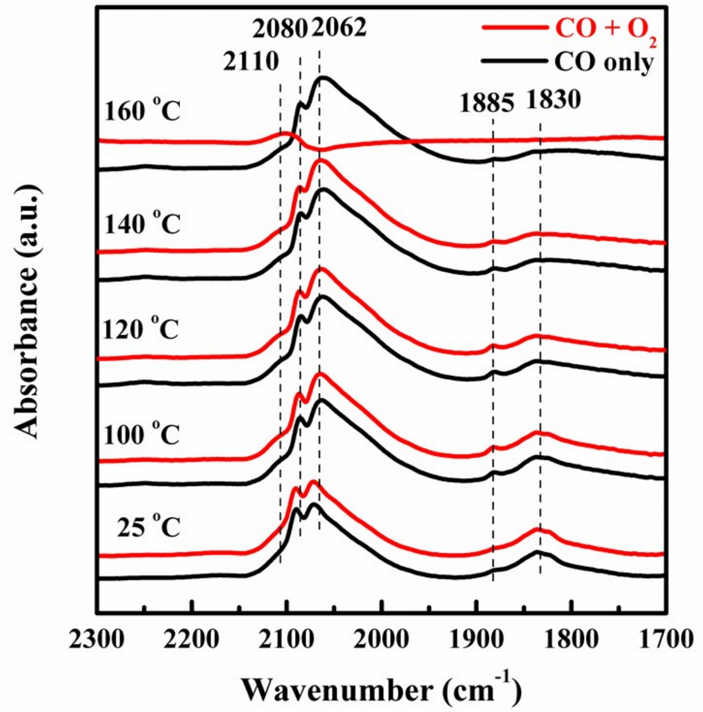

Fig. S13 The *in situ* DRIFTS spectra of CO adsorption (black lines) on Pt/Al_2O_3 catalyst at different reaction temperature. The red lines are recorded after the reaction feed gas (1% vol. CO and 2% vol. O_2) introduced into the DRIFTS cell. The peaks at 1830 cm^{-1} (1885 cm^{-1}), 2062 cm^{-1} (2080 cm^{-1}) and 2110 cm^{-1} are assigned to the CO molecules adsorbed at the bridge sites, the a-top sites, and the Pt ions or single atoms, respectively.

Fig. S14

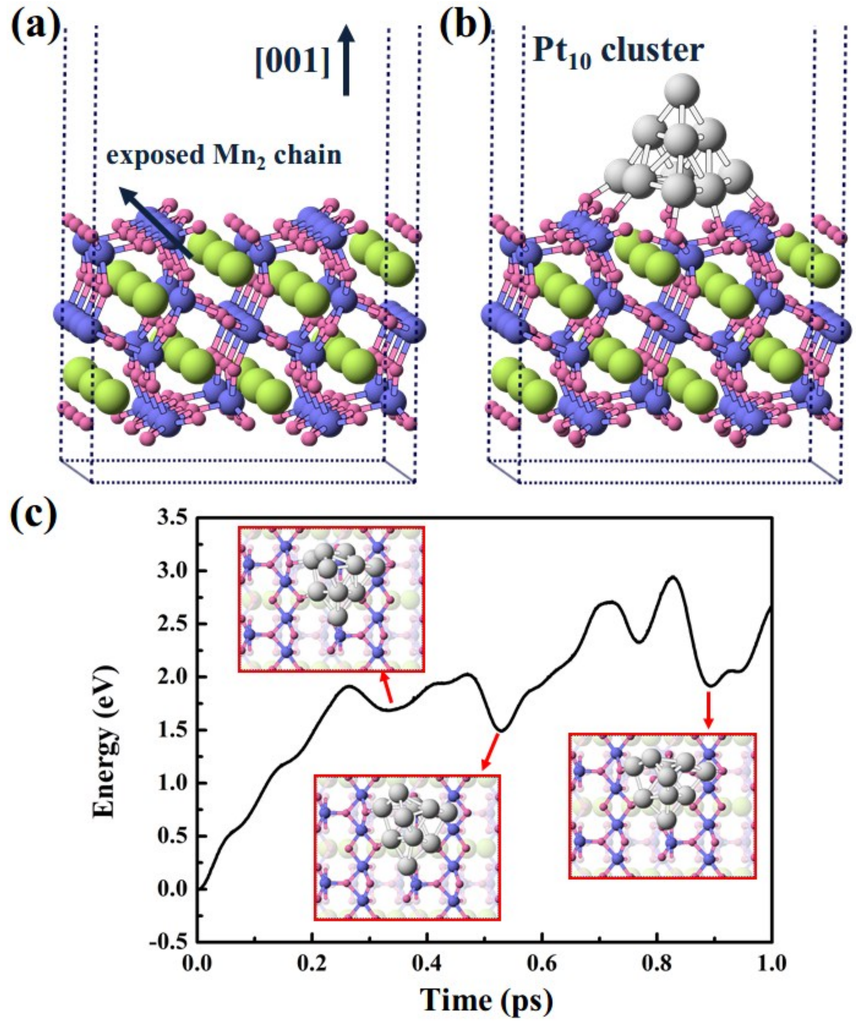

Fig. S14 The atomic structures of (a) SMO (010) slab and (b) Pt_{10}/SMO. The green, blue, pink and grey spheres represent Sm, Mn, O and Pt atoms, respectively. (c) is the total energy of Pt_{10}/SMO as a function of simulation time during the first-principle molecular dynamic simulations at 973 K. The inserted figures are the snapshots of Pt_{10}/SMO with the local minimum total energies, which indicate the structural stability of supported Pt_{10} cluster.

Fig. S15

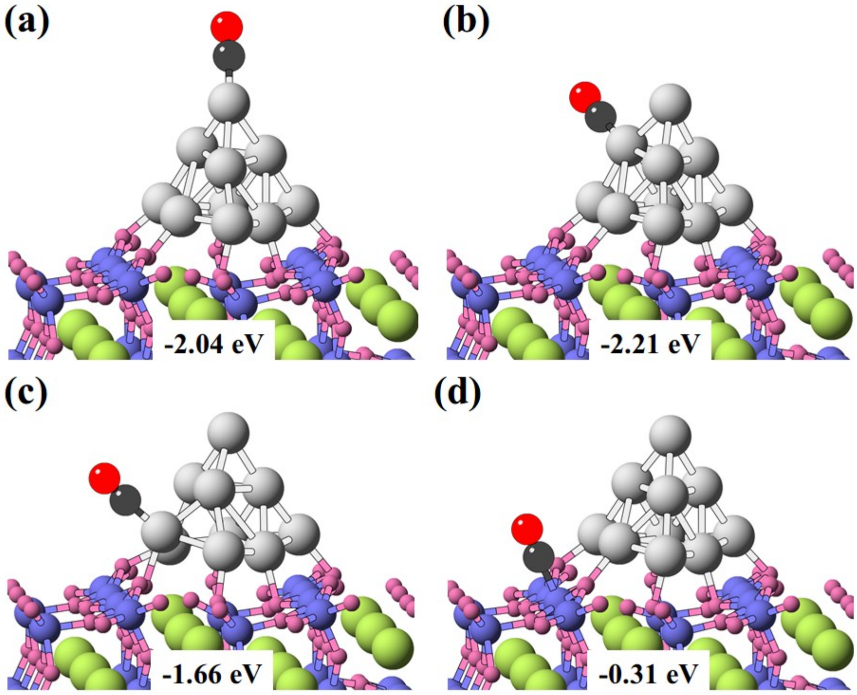

Fig. S15 The atomic structures of CO adsorption on Pt_{10}/SMO. The corresponding CO adsorption energies (E_{CO}^{ads}) are labelled. E_{CO}^{ads} is calculated by $E_{CO}^{ads} = E_{slab+CO}$ - E_{slab} - E_{CO}, where $E_{slab+CO}$, E_{slab} and E_{CO} are the total energies of Pt_{10}/SMO with adsorbed CO, pure Pt_{10}/SMO and isolated CO molecule, respectively. The results indicate that CO molecules tend to poison the Pt atoms far away from the interface with E_{CO}^{ads} smaller than -2 eV.

Fig. S16

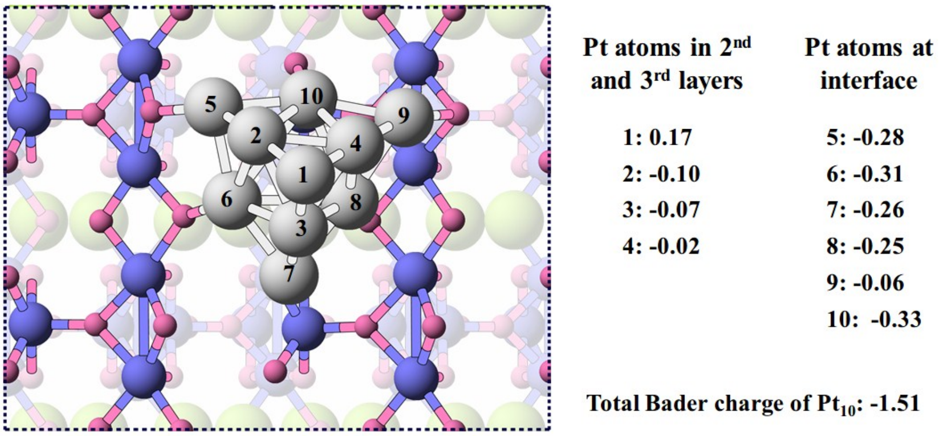

Fig. S16 The Bader charge[15] analysis of Pt_{10}/SMO. The result shows that the supported Pt_{10} cluster quantitatively donates in total 1.51*e* to the SMO slab, which are mostly from the Pt atoms at the interface.

Fig. S17

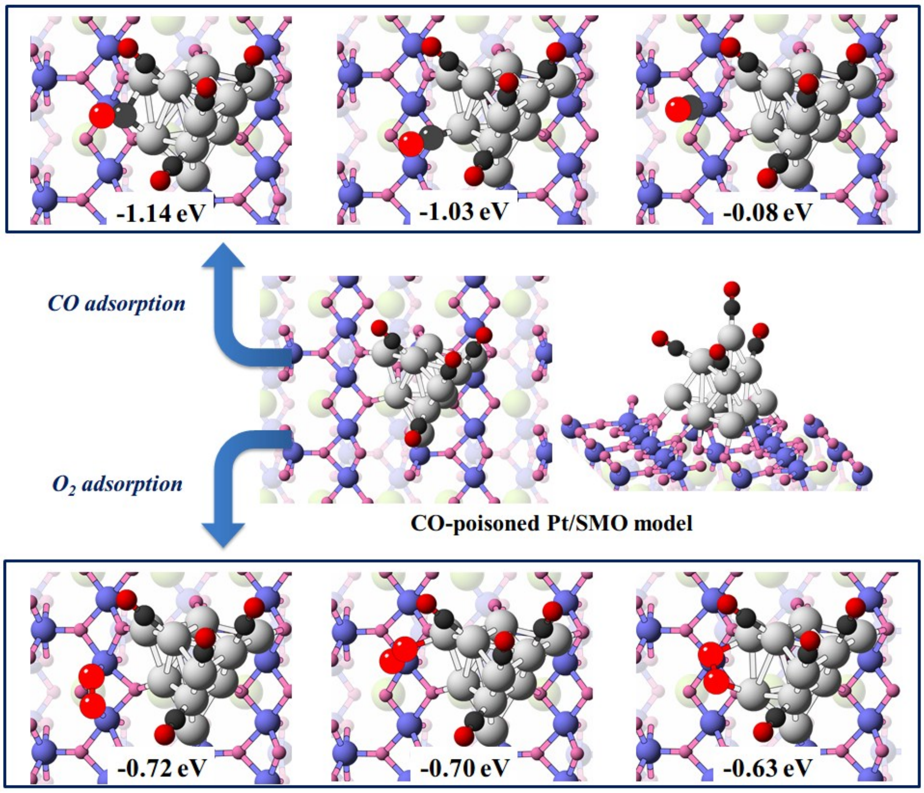

Fig. S17 Top view and side view of CO-poisoned Pt/SMO model. The atomic structures of CO and O_2 adsorption at the interface are presented, which are labelled by the corresponding adsorption energies. The results indicate that CO molecule prefers to bridged adsorb on Pt atoms at the interface (-1.14 eV), while O_2 molecule tends to adsorb on the Mn_2 dimer with the adsorption energy ($E_{O_2}^{ads}$) of -0.72 eV. $E_{O_2}^{ads}$ is calculated by $E_{O_2}^{ads} = E_{slab+O_2} - E_{slab} - E_{O_2}$, where E_{slab+O_2}, E_{slab} and E_{O_2} are the total energies of slab with adsorbed O_2, pure slab and isolated O_2 molecule, respectively.

Table S1

Table S1. Lists of the reaction temperature (T, K), CO conversion (X_{CO}), contents of Pt in mole (n_{Pt}, 10^{-6} mol), average size of Pt clusters (d_{Pt}, nm), surface Pt atoms percentage (w_{surf}), turn over frequency (TOF, 10^{-2} s^{-1}) and activation energy (E_a, kJ/mol).

Catalysts	T (K)	X_{CO} (%)	n_{Pt} (10^{-6} mol)	d_{Pt} (nm)	w_{surf}	TOF (10^{-2} s^{-1})	E_a (kJ/mol)
Pt/SMO	313	2.38	4.74	0.75	0.92	0.36	43.87
	323	4.20				0.62	
	333	6.95				0.99	
	343	11.40				1.58	
Pt_{IWI}/SMO	433	2.23	3.41	0.89	0.88	0.35	68.21
	453	3.42				0.52	
	463	5.21				0.78	
	473	7.71				1.13	
Pt/Al_2O_3	373	0.64	3.15	1.19	0.51	0.22	94.58
	383	1.60				0.37	
	393	3.50				1.15	
	403	6.70				2.15	

Table S2

Table S2. Structural information and fitting parameters obtained from Pt L_{III}-edge EXAFS of Pt/SMO and PtO_2. The fitting results of PtO_2 references agree well with the previous studies.[16,17]

sample	shell	N	R (Å)	σ^2 (Å^2)	ΔE_0 (eV)
Pt/SMO	Pt-O	3.51 ± 0.52	2.007 ± 0.014	0.0016 ± 0.0018	11.98 ± 1.78
PtO_2	Pt-O	6	2.018 ± 0.008	0.0023 ± 0.0009	10.24 ± 1.02

Table S3

Table S3. Comparing the Pt mass loading (wt%), gas hourly space velocity (GHSV, mL g^{-1} h^{-1}), 50% CO conversion temperature (T_{50}, ºC), turn over frequency (TOF, s^{-1}) and activation energy (E_a, kJ/mol) of our reported catalysts to that in previous studies. Note that the listed TOF is tested under the temperature in parentheses.

catalysts	Loading (wt%)	GHSV (mL g^{-1} h^{-1})	T_{50} (ºC)	TOF (s^{-1})	E_a (kJ/mol)	Ref.
Pt/SMO	1.85	120000	86	0.033 (90)	43.87	This work
Pt/Al_2O_3	1.23	120000	157	<0.001 (90)	94.58	This work
Pt_{IWI}/SMO	1.33	120000	240	0.011 (200)	68.21	This work
Pt/Al_2O_3	1.0	18837	176	0.014 (200)	/	[17]
Pt/Al_2O_3	10	60000	177	/	/	[18]
Pt/Al_2O_3	1.5	15000	/	0.034 (130)	115.5	[19]
Pt/TiO_2	1.0	20400	61	0.002 (27)	49	[20]
Pt/CeO_2	/	/	/	0.18 (200)	145.2	[21]
Pt/CeO_2	0.5	/	182	0.60 (200)	63.7	[22]
Pt/Co_3O_4	0.38	/	130	443 (200)	52.5	[22]
Pt/MnO_2	0.5	/	227	0.53 (200)	58.7	[22]
Pt/NiO	0.5	/	187	1.12 (200)	80.3	[22]
Pt/Fe_2O_3	0.5	/	207	0.15 (200)	59.6	[22]
Pt/FeOx	1.5	15000	/	0.151(27)	30.4	[19]

Table S4

Table S4. Comparing the adsorption energies of CO (E_{CO}^{ads}, eV) and O_2 ($E_{O_2}^{ads}$, eV), as well as the barrier energies of O_2 dissociation ($E_{O_2}^{dis}$, eV) and CO_2 formation ($E_{CO_2}^{form}$, eV) of our proposed reaction route at the interface of CO-poisoned Pt_{10}/SMO to references.

	E_{CO}^{ads} (eV)	$E_{O_2}^{ads}$ (eV)	$E_{O_2}^{dis}$ (eV)	$E_{CO_2}^{form}$ (eV)	Ref.
Pt_{10}/SMO	-1.14	-0.46	0.41	0.22	This work
Pt_{11}/TiO_2	-1.39	-1.68	0.52	0.98	Ref. 23
Pt_{14}/TiO_2	-1.80	-1.49	0.64	0.62	Ref. 24
Pt_4/CeO_2	-2.02	/	/	/	Ref. 25
Pt_4/CeO_2	/	-1.39	1.93	/	Ref. 26
$Pt_4/La_{0.625}Sr_{0.375}Co_{0.25}Fe_{0.75}O_{3-\delta}$	/	-1.89	0.53	/	Ref. 27
Pt_{10}/Al_2O_3	-2.13	-1.60	0.60	0.30	Ref. 28
Isolated Pt_{10}	-2.07	-0.81	0.60	0.30	Ref. 28

References

[1] W. C. Wang, G. McCool, N. Kapur, G. Yuan, B. Shan, M. Nguyen, U. M. Graham, B. H. Davis, G. Jacobs, K. Cho and X. H. Hao, *Science*, 2012, **337**, 832-835.

[2] Z. J. Feng, J. Q. Wang, X. Liu, Y. W. Wen, R. Chen, H. F. Yin, M. Q. Shen and B. Shan, *Catal. Sci. Technol.*, 2016, **6**, 5580-5589.

[3] X. Liu, Q. Q. Zhu, Y. Lang, K. Cao, S. Q. Chu, B. Shan and R. Chen, *Angew. Chem. Int. Ed.*, 2017, **56**, 1648-1652.

[4] B. Ravel and M. J. Newville, *Synchrotron Rad.*, 2005, **12**, 537-541.

[5] A. D. Allian, K. Takanabe, K. L. Fujdala, X. H. Hao, T. J. Truex, J. Cai, C. Buda, M. Neurock and E. Iglesia, *J. Am. Chem. Soc.*, 2011, **133**, 4498-4517.

[6] G. Kresse and J. Hafner, *Phys. Rev. B*, 1993, **47**, 558-561.

[7] G. Kresse and J. Hafner, *Phys. Rev. B*, 1994, **49**, 14251-14269.

[8] G. Kresse and J. Furthmüller, *Comput. Mater. Sci.*, 1996, **6**, 15-50.

[9] Y. Ishii, S. Horio, M. Mitarashi, T. Sakakura, M. Fukunaga, Y. Noda, T. Honda, H. Nakao, Y. Murakami and H. Kimura, *Phys. Rev. B*, 2016, **93**, 064415.

[10] H. B. Li, W. H. Wang, X. Y. Qian, Y. H. Cheng, X. J. Xie, J. Y. Liu, S. H. Sun, J. G. Zhou, Y. F. Hu, J. P. Xu, L. Li, Y. Zhang, X. W. Du, K. H. Gao, Z. Q. Li, C. Zhang, S. D. Wang, H. J. Chen, Y. D. Zhao, F. Lu, W. C. Wang and H. Liu, *Catal. Sci. Technol.*, 2016, **6**, 3971-3975.

[11] J. P. Perdew, K. Burke and M. Ernzerhof, *Phys. Rev. Lett.*, 1996, **77**, 3865-3868.

[12] G. Kresse and D. Joubert, *Phys. Rev. B*, 1999, **59**, 1758-1775.

[13] H. J. Monkhorst and J. D. Pack, *Phys. Rev. B*, 1976, **13**, 5188-5192.

[14] G. Henkelman and H. Jónsson, *J. Chem. Phys.*, 2000, **113**, 9978-9985.

[15] G. Henkelman, A. Arnaldsson and H. Jónsson, *Comput. Mater. Sci.*, 2006, **36**, 354-360.

[16] S. Gatla, D. Aubert, G. Agostini, O. Mathon, S. Pascarelli, T. Lunkenbein, M. G. Willinger and H. Kaper, *ACS Catal.*, 2016, **6**, 6151-6155.

[17] M. Moses-DeBusk, M. Yoon, L. F. Allard, D. R. Mullins, Z. Wu, X. Yang, G. Veith, G. M. Stocks and C. K. Narula, *J. Am. Chem. Soc.*, 2013, **135**, 12634-12645.

[18] P. Thormählen, M. Skoglundh, E. Fridell and B. Andersson, *J. Catal.*, 1999, **188**,

300-310.

[19] L. Q. Liu, F. Zhou, L. G. Wang, X. J. Qi, F. Shi and Y. Q. Deng, *J. Catal.*, 2010, **274**, 1-10.

[20] G. R. Bamwenda, S. Tsubota, T. Nakamura and M. Haruta, *Catal. Lett.*, 1997, **44**, 83-87.

[21] L. Feng, D. T. Hoang, C. Tsung, W. Y. Huang, S. H. Lo, J. B. Wood, H. Wang, J. Y. Tang and P. D. Yang, *Nano Res.*, 2011, **4**, 61-71.

[22] K. An, S. Alayoglu, N. Musselwhite, S. Plamthottam, G. Melaet, A. E. Lindeman and G. A. Somorjai, *J. Am. Chem. Soc.*, 2013, **135**, 16689-16696.

[23] Q. X. Cai, X. D. Wang and J. G. Wang, *J. Phys. Chem. C*, 2013, **117**, 21331-21336.

[24] H. Zhou, X. L. Chen and J. G. Wang, *Int. J. Quantum Chem.*, 2016, **116**, 939-944.

[25] C. Jung, H. Tsuboi and M. Koyama, *Catal. Today*, 2006, **111**, 322-327.

[26] T. Q. Nguyen, M. C. S. Escaño and H. Nakanishi, *Appl. Surf. Sci.*, 2014, **288**, 244-250.

[27] W. Q. Yang, Z. B. Wang and Z. Q. Wang, *ACS Appl. Mater. Interfaces*, 2014, **6**, 21051-21059.

[28] C. R. Yin, F. R. Negreiros and G. Barcaro, *J. Mater. Chem. A*, 2017, **5**, 4923-4931.
